# Photoprotection practices, knowledge and sun-related skin damage in Spanish beach handball players

**DOI:** 10.7717/peerj.7030

**Published:** 2019-06-18

**Authors:** Guillermo De Castro-Maqueda, Jose Vicente Gutierrez-Manzanedo, Carolina Lagares-Franco, Mario Linares-Barrios, Magdalena de Troya-Martin

**Affiliations:** 1 Physical Education Department, Education Science Faculty, Universidad de Cádiz, Cádiz, Spain; 2 Departament of Statistics and Operational Research, Universidad de Cádiz, Cádiz, Spain; 3 Dermatology Departament, Puerta del Mar University Hospital, Cádiz, Spain; 4 Dermatology Departament, Costa del Sol Hospital, Marbella, Málaga, Spain

**Keywords:** Skin cancer, Photoprotection, Skin damage, Beach handball

## Abstract

**Background:**

Outdoor sports are a risk activity for skin cancer, especially if adequate sun protection measures are not used. The aim of this study is to examine the photoprotection habits of outdoor (beach) handball players, and to determine the relation between duration of sports practice, photoprotection behaviour and sun-related damage to the skin.

**Methods:**

This cross-sectional study is based on a health survey of sun exposure and protection habits and practices conducted among beach handball players in southern Spain. This survey provided data for a descriptive and comparative analysis, by groups and gender, of photoprotection and skin self-examination practices.

**Results:**

Among the whole sample, 76.9% had suffered at least one sunburn event during the last year. By groups, 73.97% of the older participants (Group I, University students) and 81.25% of the younger ones (Group II, youngers players) reported this outcome, and the difference was statistically significant (*p* = 0.003). With respect to photoprotection, 68.5% of the players in group I and 66.7% of those in group II used sun cream with a protection factor of 30 or higher, although 52.1% of group I and 35.4% of group II did not reapply it. As concerns self-examination, 94.5% of group I and 87.5% of group II had not examined their body for skin damage during the previous year. Medical examination revealed the presence of lentigines and freckles among many players, with no significant differences between the two groups.

**Conclusions:**

Beach handball players are highly exposed to the effects of ultraviolet radiation and often take insufficient measures of sun protection. Programmes should be designed and implemented to raise awareness among adolescent and young adult sport competitors of the risks of skin cancer associated with their sports activity and to encourage them to improve their photoprotection and skin monitoring practices.

## Introduction

The incidence of skin cancer (melanoma, basal cell carcinoma and squamous cell carcinoma) is increasing at a rate higher than that of any other malignancy, worldwide, (Leiter, Eigentler & Garbe, 2014) and exposure to ultraviolet radiation is the main preventable cause of skin cancer. Two patterns of solar risk exposure have been identified: the first is occupational, chronic and cumulative, and related to an increased risk of squamous cell carcinoma; the second is recreational, acute and intermittent, and associated with an increased risk of melanoma and basal cell carcinoma (Armstrong & Kricker, 2001; Molho-Pessach & Lotem, 2007). Sun-related damage that occurs during the first years of life plays a decisive role in the development of skin cancer in later life. Sunburn in childhood and adolescence is the main risk factor for melanoma (Armstrong, 2004).

Up to 80% of recorded cases of skin cancer could be prevented by reducing sun exposure and through the use of protective measures against ultraviolet radiation, such as sunscreen cream, headwear, sunglasses and long-sleeved shirts, when activities are carried out outdoors on sunny days (Stanton et al., 2004).

The means of photoprotection most commonly adopted, by all age groups, is the use of sunscreen (Stanton et al., 2004). However, many people apply it insufficiently or incorrectly, thus increasing their exposure to the sun and heightening the risk of sunburn (Hall et al., 2001; Autier, Boniol & Dore, 2007). Epidemiological studies have shown that the use of photoprotective cream significantly reduces the risk of actinic keratosis and squamous cell carcinomas (Green et al., 1999). Another relevant factor is that of education background, which can be a significant predictor of sunburn. People with secondary or university education report the highest number of sunburn events, due to the social pressure of the aesthetics of tanning (De Troya-Martin et al., 2018). Moreover, although there are differences between adults, adolescents and children regarding sun exposure, most surveys in this area have shown that very few people protect themselves sufficiently from the sun and therefore the prevalence of sunburn tends to be very high (Dobinsson & Hill, 2004; Toro-Huamanchumo et al., 2019). Most research in this field is based on health surveys measuring self-reported behaviour concerning exposure habits and photoprotection practices during outdoor sports, focused on skaters (Fernandez-Morano et al., 2017), cyclists (Petty, Knee & Joseph, 2013), surfers, tennis, hockey and football players (Lawler et al., 2007) and aquatics athletes (De Castro-Maqueda et al., 2019).

New outdoor sports at the beach such as beach handball are growing popularity among young people as shown by the increase in participation in youth championships events (International Handball Federation, 2018). Therefore, many young people spend long periods of time outdoors and usually without adequate protection from the sun. The radiation they receive while playing on the beach is particularly high, because the direct radiation of the sun’s rays is reflected by the surfaces of seawater and sand. In addition, sweat reduces the effectiveness of sun cream. In consequence, beach handball players are at particularly high risk of sunburn and, in the future, of skin cancer and accelerated aging of the skin, being likely to suffer changes in skin pigmentation and the early appearance of wrinkles in exposed areas.

The aim of the present study is to examine the photoprotection habits of beach handball players and to determine the association between the time spent playing outdoors, the protective measures adopted and the sun damage apparent on their skin.

## Materials & Methods

This cross sectional study is based on a health survey of sun exposure and protection habits and practices conducted among beach handball players in southern Spain. After the interviews, a total cutaneous examination using dermoscopy and Wood lamp illumination was performed by a dermatologist to identify potentially malignant lesions.

All data were recorded anonymously and treated in strict compliance with Spanish data protection laws (Law 41/2002 of 14 November and Law 15/1999 of 13 December).

### Participants and eligibility criteria

The study group was selected by convenience sampling, among beach handball players at the Spanish University Championships held in Cádiz (La Victoria beach) in May 2017. Participation was voluntary, and the only requirements were the provision of informed consent by players aged 18 years or more, and that of their parents for younger players, and having an adequate understanding of written or spoken Spanish. After answering the survey and taking the medical examination, the participants received an informative brochure on sun protection and were given samples of sunscreen creams.

The following inclusion criteria were applied: beach handball players aged 12–30 years, participating in the University Championships or playing for a federated club in the province of Cádiz.

The study protocol was reviewed and approved by the institutional review board where the investigators were then employed, namely the Medical Ethics Committee of the Dermatology Service, Puerta del Mar University Hospital, Cádiz and the Department of Physical Education, School of Education, University of Cádiz (UCA13/0913). All participants (or their parents) provided written informed consent before the start of this study.

### Questionnaire

The survey document was a self-administered questionnaire with measures adapted and/or selected from two questionnaires of previous surveys regarding sun exposure and sun protection practices (Glanz et al., 2008) and knowledge of exposure to the sun (De Troya-Marín et al., 2009). The questionnaire by Glanz et al. (2008) showed a good clarity and applicabilitity for measuring sun exposure and sun protection behaviors (Glanz et al., 2008). This instrument has been used in previous studies (De Castro-Maqueda et al., 2019; Glanz et al., 2008; Glanz et al., 2010). The questionnaire by De Troya-Marín et al. (2009) has been showed to be valid and reliable with intraclass correlation and d coefficient values >0.70 (De Troya-Marín et al., 2009). This tool has also been used in earlier studies (De Troya-Marín et al., 2009; De Troya-Martin et al., 2018). The questions were designed to obtain information on demographic variables (age, gender, nationality) and on the players’ sun exposure habits and knowledge. In addition, a form recording the results of a skin status examination and epidemiological record was completed by the attending dermatologist, to document any skin damage observed.

The questionnaire was designed to be completed in 5–10 min, in the presence of the interviewer, to resolve any doubts that might arise and to ensure the procedure was performed consistently. For adults, the interviewer that was present only interact when questioned; for teenagers, interviewer only read the questions taking care not to interfere in the answers.

The questionnaire included the following three sections:

### Part 1: Sun exposure habits

#### Section 1

##### Sun habits.

(a) Average time spent in the sun between 10 a.m. and 4 p.m., on weekdays (30 min to 6 h); (b) Average time spent in the sun between 10 a.m. and 4 p.m. at the weekend (30 min to 6 h). (c) Sunburn events during the previous year. Answers scored on a range from 0 to “5 or more” (Glanz et al., 2008). Sunburn was defined as the presence of blistering and/or reddening and/or pain lasting more than one day (Glanz et al., 2008).

*Sun protection practices* (items recommended by the World Health Organization, 2017): Use sunscreen, wear long-sleeved T-shirts, wear headgear, stay in the shade, and wear sunglasses. As a risk practice, participants were asked about their sun exposure performed in order to achieve tanned skin (Glanz et al., 2008). All responses were registered on a 4-point likert scale (1, Rarely or never; 2, Sometimes; 3, Often; 4, Always).

*Skin colour*: Colour of skin not exposed to sunlight (6 response categories: very fair; fair; very light; olive; medium; very dark) (Fitzpatrick, 1988).

#### Section 2

##### Skin check-up.

The players were asked if they had ever had a medical skin check-up (yes/no), and if so, when the last time had been (month and year).

They were also asked if they themselves or someone else had examined their skin, including their back, in the past year, to search for spots or lesions. If the answer was yes, the number of times such an examination had been made was also recorded.

#### Section 3

##### Training habits.

The following questions were prepared by a group of experts in physical education and sports, and included in the questionnaire.

a. - On average, how many hours do you train outdoors each day?

b. - When you are training or competing, do you usually put sunscreen on your face? If so, what sun protection factor does the sunscreen have?

c. - Do you usually reapply sunscreen throughout the day?

### Part 2: Knowledge about sun exposure

These questions determine the participants’ basic knowledge about sun exposure and skin cancer. All should be answered as ‘true’ or ‘false’ (De Troya-Marín et al., 2009).

1. Sunscreen prevents the skin aging produced by solar radiation.
2. The sun is the main cause of skin cancer.
3. Exposure to the sun provokes spots on the skin.
4. If I use barrier cream, I can expose my skin to the sun without risk.
5. Avoiding exposure to the sun during the central hours of the day (11 a.m. to 5 p.m.) is the most effective way to protect the skin from the sun.
6. Avoiding exposure to the sun during childhood and adolescence reduces the risk of skin cancer by 80%.
7. Once my skin goes brown, I won’t need to use sunscreen.

### Part 3: Skin examination record form

Each player was examined by a dermatologist for sun-related skin damage and the following aspects were recorded:

a.- Fitzpatrick skin type (I–VI) (Fitzpatrick, 1988):

Phototype I: Pale white skin, blue/green eyes, blond/red hair. Always burns, does not tan.

Phototype II: Fair skin, blue eyes. Burns easily, tans poorly.

Phototype III: Darker white skin. Tans after initial burn.

Phototype IV: Light brown skin. Burns minimally, tans easily.

Phototype V: Brown skin. Rarely burns, tans darkly easily.

Phototype VI: Dark brown or black skin. Never burns, always tans darkly.

b.- Presence of sun-related damage, such as lentigines, actinic keratosis, actinic cheilitis, telangiectasia or freckles. (Yes/No)

c.- Skin colour, exposed and non-exposed to the sun (Light/Dark)

d.- Eye colour (1. Black, 2. Brown, 3. Green, 4. Grey, 5. Blue, 6. Other)

e.- Hair colour (1. Black, 2. Brown, 3. Blonde, 4. Red)

f.- Change in hair colour at 20–30 years (Yes/No)

g.- Glogau Classification of Photo-aging (I–IV):

Type I: no wrinkles, incipient photo-aging, slight pigmentary changes, no keratosis, minimal wrinkles, age 25–30 years.

Type II: wrinkles in motion, moderate photo-aging, incipient senile lentigines, palpable but non-visible keratosis, incipient parallel smile lines, age 35–50 years.

Type III: wrinkles at rest, advanced photo-aging, dyschromias and telangiectasias, visible keratosis, age 50–60 years.

Type IV: wrinkles throughout, intense photo-aging, yellowish-grey skin colour, pre-cancerous skin, generalised wrinkles with abnormal skin, age 60–80 years.

The questionnaire also includes some items used in previous studies in this field, such as the re-application of sunscreen and its protection factor (Glanz & Mayer, 2005; Glanz et al., 2008), the Fitzpatrick skin type (Fitzpatrick, 1988), the use of sunscreen, history of sunburn events, previous medical examinations of the skin, self-examination of the skin. All the interviewees were beach handball players; some were taking part in the Spanish University Championships and others were playing in beach handball teams based in the province of Cádiz; the latter were aged under 18 years and, rather than completing the questionnaire themselves, responded verbally to trained interviewers during the Championships (Glanz & Mayer, 2005).

### Statistical analysis

This descriptive and comparative analysis by groups according to competitive categories focuses on the participants’ photoprotection and skin self-examination habits. The chi-square test was used when qualitative variables were crossed. For quantitative variables crossed with qualitative variables, the Kolmogorov–Smirnov test of normality was applied. When normal distribution was observed, the Student t test was applied, and otherwise the Wilcoxon test. For chi-square analyses, the effect sizes (ES) was measurement with Cramér’s V. The magnitude of the effect size statistics was considered negligible 0.00–0.10; weak 0.10–0.20; moderate, 0.20–0.40; relatively strong, 0.40–0.60; strong, 0.40–0.80; very strong, 0.80–1.00 (Lee, 2016). In all cases a significance level of 5% is assumed. All analyses were performed using IBM SPSS v.22 statistical software.

## Results

A total of 129 players participated in this study. Of these, 5 were excluded as the parental consent were not provided. The questionnaires were answered by 124 players. After analysing the questionnaire responses, three participants were excluded because one or more questions was not answered. The final sample involved 121 beach handball players. The study sample was divided into two groups depending on the category in which the participants competed: group I, composed of university students (*n* = 73, 40 men, 54.8% and 33 women, 45.2%) and group II, composed of younger players (*n* = 48, 41, and 85.4% boys and 7 girls, 14.6%). The physical characteristics of both groups are shown in Table 1.

**Table 1:** Physical characteristics and playing experience.

	Group I (*n* = 73)			Group II (*n* = 48)		
Variables	Men	Women	Total_GroupI_	Boys	Girls	Total_GroupII_
Age (years)	21.95 (2.76)	22.36 (3.40)	22.14 (3.05)	13.07 (2.48)	11.43 (1.40)	12.83 (2.42)
Playing experience (years)	4.38 (4.50)	3.06 (2.30)	3.78 (3.70)	1.78 (0.91)	1.14 (0.38)	1.69 (0.88)
Weight (kg)	79.23 (9.66)	61.55 (7.17)	71.23 (12.32)	57.24 (14.6)	42.57 (8.66)	55.10 (14.78)
Height (cm)	180.15 (7.75)	166.15 (7.34)	173.82 (10.28)	159.37 (15.42)	151.43 (11.69)	158.21 (15.09)

**Notes.**

Results are expressed as Mean (standard deviation).

Among the players in both groups, 57.5% had less than two hours of sun exposure from Monday to Friday, and 31.5% during the weekend. However, 76.9% had suffered at least one sunburn with consequences lasting more than a day during the previous year. By groups, 73.97% of group I and 81.25% of group II had suffered at least one sunburn during the previous year and the difference was statistically significant (*p* = 0.003). With respect to their sun exposure on hot summer days, the groups differed in their use of sunscreen, headwear and sunglasses and in their wish to become tanned (Table 2).

**Table 2:** Sun protection habits on hot summer days.

Variables	Group I				Group II				Effect size	Sig.
	Rarely or never *n* (%)	Sometimes *n* (%)	Often *n* (%)	Always *n* (%)	Rarely or never *n* (%)	Sometimes *n* (%)	Often *n* (%)	Always *n* (%)		
Use sunscreen	4 (5.5)	30 (41.1)	26 (35.6)	13 (17.8)	9 (18.8)	11 (22.9)	13 (27.1)	15 (31.3)	0.294	0.015
Wear a long-sleeved shirt	38 (52.1)	13 (17.8)	18 (24.7)	4 (5.5)	21 (43.8)	12 (25)	10 (20.8)	5 (10.4)	0.137	0.52
Wear a hat/cap	52 (71.2)	15 (20.5)	6 (8.2)	–	42 (87.5)	2 (4.2)	4 (8.3)	–	0.232	0.038
Seek the shade	12 (16.4)	30 (41.1)	23 (31.5)	8 (11)	9 (18.8)	15 (31.3)	14 (29.2)	10 (20.8)	0.152	0.43
Wear sunglasses	16 (21.9)	15 (20.5)	22 (30.1)	20 (27.4)	24 (50)	13 (27.1)	9 (18.8)	2 (4.2)	0.380	0.001
Sunbathe in order to tan	16 (21.9)	18 (24.7)	23 (31.5)	16 (21.9)	32 (66.7)	8 (16.7)	5 (10.4)	3 (6.3)	0.460	0.000

The most common skin colour was light brown (Group 1, 28 players; Group II, 32 players). By Fitzpatrick skin type, 25 players in Group I were type III and 48 were type IV. In Group II, 20 players were type III and 28 players were type IV.

Very few conducted self examination of the skin; thus, 94.5% of those in Group I and 87.5% of those in Group II had not examined their body for skin lesions during the last year.

In sports practice and training, there were significant differences between the groups (*p* = 0.002), with the players in Group II spending significantly more time in this respect. Approximately half of the players in each group did not apply sunscreen when training or competing (Group I, 49.3%; Group II, 56.3%). Among those who did, 68.5% of Group I and 66.7% of Group II used sunscreen with a protection factor of 30 or higher, but 52.1% of Group I and 35.4% of Group II did not reapply it.

With respect to knowledge about sun exposure, there were significant differences between the groups (see Table 3).

**Table 3:** Knowledge about sun exposure.

	Group I		Group II		Effect size	Sig.
	True	False	True	False		
	*n* (%)	*n* (%)	*n* (%)	*n* (%)		
Sun protection creams prevent aging of the skin produced by solar radiation	69 (94.5)	4 (5.5)	31 (64.4)	17 (35.4)	0.387	0.000
The sun is the main cause of skin cancer	71 (97.3)	2 (2.7)	41 (85.4)	7 (14.6)	0.221	0.028
The sun produces marks on the skin	71 (97.3)	2 (2.7)	42 (87.5)	6 (12.5)	0.192	0.057
If I use total sun block I can sunbathe without any risk	5 (6.8)	68 (93.2)	14 (29.2)	34 (70.8)	0.300	0.001
Avoiding the midday sun (11–17 h.) is the most efficient way of protecting my skin	66 (90.4)	7 (9.6)	37 (77.1)	11 (22.9)	0.183	0.044
Avoiding the sun at young ages (under 18 years) reduces the risk of skin cancer by 80%	53 (72.6)	20 (27.4)	24 (50)	24 (50)	0.230	0.011
Once my skin is tanned, I don’t need to use sun protection cream	2 (2.7)	71 (97.3)	12 (25)	36 (75)	0.340	0.000

**Notes.**

*p*-values reflect significant differences between the groups.

Evidence of sun damage to the skin was basically in the form of lentigines and freckles (Table 4). In this respect, there were no significant differences between the groups. The results for the Glogau photo-aging scale showed that 97.3% of the players in Group I and 100% of those in Group II were type I, reflecting the youth of the participants.

**Table 4:** Signs of sun-related skin damage.

	Group I		Group II	
	**YES n (%)**	**NO n (%)**	**YES n (%)**	**NO n (%)**
Lentigines	31 (42.5)	42 (57.5)	27 (56.3)	21 (43.8)
Actinic keratosis	–	73 (100)	–	48 (100)
Actinic cheilitis	1 (1.4)	72 (98.6)	–	48 (100)
Telangiectasia	–	73 (100)	–	48 (100)
Freckles	24 (32.9)	49 (67.1)	11 (22.9)	37 (77.1)

## Discussion

This study was conducted to examine the sun-related habits and knowledge of beach handball players. The participants completed a questionnaire in this respect, and the information obtained was complemented by a dermatological examination, to identify sun-related damage. Analysis revealed inadequate photoprotection habits among these players, which is worrying in view of the growing popularity of outdoor sports and hence participants’ increased exposure to UV rays and greater risk of suffering skin lesions.

The majority of the handball players in this study reported having suffered a sunburn event during the previous season, either in competition or in training, which is in line with research findings for players of other sports (Bränstrom et al., 2010; De Castro-Maqueda et al., 2019; Fernandez-Morano et al., 2017). However, the participants in our study reported higher rate of sunburn compared with other populations ( De Troya-Martin et al., 2016; De Troya-Martin et al., 2018; Toro-Huamanchumo et al., 2019).

Our study shows that age is an important factor. The younger players were more likely to suffer sunburn, with 45.8% reporting having suffered two or more burns during the last season, which is comparable with the high rates of sunburn among adults and adolescents reported in previous studies (Davis et al., 2002; Fernandez-Morano et al., 2014; Lawler et al., 2007).

Younger players make significantly less use of sunscreen than university students. Thus, 56.3% and 49.3%, respectively, never use sunscreen on the face when training/competing. Nevertheless, these figures are higher than those found elsewhere; in the USA, only 15% of university-age athletes use sunscreen. Among other reasons suggested for the low level of use of sunscreen, the participants in our study mentioned forgetfulness and discomfort (Fernandez-Morano et al., 2014). Nevertheless, a previous study, based on young people, reported that young athletes make significantly more use of sunscreen than non-athletes (Lawler et al., 2012).

With respect to the reapplication of sunscreen, the results differed according to age. Thus, 52.1% of the university-age players did not reapply sun protection cream, compared to 35% of the beginners. This question is of great importance, as the reapplication of sunscreen is almost as important as the first use, and it should be reapplied after 30 min to ensure adequate protection (Hamant & Adams, 2005).

Two-thirds of the total sample used sunscreen with a protection factor of 30 or more, which is a better result than has been found for skaters, among whom only 20% use creams with a protection factor greater than 15 (Cohen, Tsai & Puffer, 2006; Jinna & Adams, 2013).

Although the participants spend many hours exposed to the sun in training and competition, this time is slightly less than that recorded for other athletes such as surfers or skaters (Diffey, 2001; Fernandez-Morano et al., 2017; De Castro-Maqueda et al., 2019). With respect to the use of shade to protect the skin from the sun, our results are similar to those reported for practitioners of other outdoor sports (Lawler et al., 2007; Petty, Knee & Joseph, 2013).

Our analysis showed there were significant differences in the use of sunglasses between the two study groups, a finding we consider to be normal, as the players in Group II are very young, and so do not usually wear glasses. On the other hand, we were unable to compare our results with those for other sports, because in many cases players are not allowed to wear sunglasses during competition, for safety reasons.

With respect to knowledge about the sun and its impact on the skin, research has shown that although athletes with fewer educational qualifications report a greater awareness of cancer, those with more education have a higher incidence of melanoma (Clegg et al., 2009).

The findings of this study show the need to improve photoprotection practices, through educational interventions that motivate young people to use photoprotective suncream (Pagoto, McChargue & Fuqua, 2003) and to learn how to apply it correctly (20 min before sun exposure, in appropriate quantity -2 mg/cm2- and reapply every 2 h and after bathing) to ensure its effectiveness (Wright, Wright & Wagner, 2001).

## Conclusions

In conclusion, the results of this study indicate that beach handball players are exposed to potentially harmful UV rays and that many were affected by sunburn during the last playing season. Therefore, it is necessary to raise awareness among this population of the dangers faced in this respect and of the direct relationship between excessive exposure to the sun and the development of skin cancer. A change is needed, both in exposure habits and in protection attitudes, via awareness campaigns and education in healthy lifestyles, especially for younger players. Finally, parents, trainers and educators should teach and demonstrate good skin care practices.

## Additional Information and Declarations

### Data Availability

The following information was supplied regarding data availability: Data is available at Figshare: de Castro Maqueda, Guillermo (2019): Beach Hand Ball.sav. figshare. Dataset. https://doi.org/10.6084/m9.figshare.7731563.v1

## References

[ref-1] Armstrong BK (2004). How sun exposure causes skin cancer: an epidemiological perspective. Prevention of skin cancer.

[ref-2] Armstrong BK, Kricker A (2001). The epidemiology of UV-induced skin cancer. Journal of Photochemistry and Photobiology B, Biology.

[ref-3] Autier P, Boniol M, Dore JF (2007). Sunscreen use and the increased duration of intentional sun exposure: still a burning issue. International Journal of Cancer.

[ref-4] Bränstrom R, Kasparian NA, Chang YM, Affleck P, Tibben A, Aspinwall LG, Azizi E, Baron-Epel O, Battistuzzi L, Bergman W, Bruno W, Chan M, Cuellar F, Debniak T, Pjanova D, Ertmanski S, Figl A, Gonzalez M, Hayward NK, Hocevar M, Kanetsky PA, Leachman SA, Heisele O, Palmer J, Peric B, Puig S, Schadendorf D, Gruis NA, Newton-Bishop J, Brandberg Y (2010). Predictors of sun protection behaviors and severe sunburn in an international online study. Cancer Epidemiology Biomarkers & Prevention.

[ref-5] Clegg LX, Reichman ME, Miller BA, Hamkey BF, Singh GK, Lin YD, Goodman MT, Lynch CF, Schwartz SM, Chen VW, Bernstein L, Gomez SL, Graff JJ, Lin CC, Johnson NJ, Edwards BK (2009). Impact of socioeconomic status on cancer incidence and stage at diagnosis: selected findings from surveillance, epidemiology, and end results: National Longitudinal Mortality Study. Cancer Causes Control.

[ref-6] Cohen PH, Tsai H, Puffer JC (2006). Sun-protective behavior among high-school and collegiate athletes in Los Angeles. Clinical Journal of Sport Medicine.

[ref-7] Davis KJ, Cokkinides VE, Weinstock MA, O’Connell MC, Wingo PA (2002). Summer sunburn and sun exposure among US youths aged 11 to 18: national prevalence and associated factors. Pediatrics.

[ref-8] De Castro-Maqueda G, Gutierrez-Manzanedo JV, Ponce-González J, Fernandez-Santos JR, Linares-Barrios M, De Troya-Martín M (2019). Sun protection habits and sunburns in aquatics elite athletes: Surf, Windsurf and Olympic sail. Journal of Cancer Education.

[ref-9] De Troya-Marín M, Blázquez-Sánchez N, Rivas Ruiz F, Fernández-Canedo I, Rupérez-Sandoval A, Pons Palliser J, Perea-Milla E (2009). Validación de un cuestionario en español sobre comportamientos, actitudes y conocimientos relacionados con la exposición solar: Cuestionario a pie de playa. Actas Dermo-sifiliograficas.

[ref-10] De Troya-Martin M, Gálvez-Aranda MV, Rivas-Ruiz F, Blázquez-Sánchez N, Fernández-Morano MT, Padilla-España L, Herrera-Ceballos E (2018). Prevalence and predictors of sunburn among beachgoers. Photodermatology Photoimmunology Photomedicine.

[ref-11] De Troya-Martin M, Padilla-España L, Fernandez-Morano T, Delgado-Sánchez N, Blázquez-Sánchez N, Rivas-Ruiz F, Herrera-Ceballos E,  De Gálvez-Aranda MV (2016). Sun protection habits and attitudes among healthcare personnel in a Mediterranean population. Journal of Cancer Education.

[ref-12] Diffey BL (2001). When should sunscreen be reapplied?. Journal American Academic Dermatology.

[ref-13] Dobinsson S, Hill D (2004). Patterns and causes of sun exposing and sun protecting behaviour. Prevention of skin cancer.

[ref-14] Fernandez-Morano T, De Troya-Martin M, Rivas-Ruiz F, Blázquez-Sánchez N, Del Boz-González J, Fernández-Peñas P, Buendía-Eisman A (2014). Behaviours, attitudes and awareness concerning sun exposure in adolescents on the Costa del Sol. European Journal Dermatology.

[ref-15] Fernandez-Morano T, De Troya-Martín M, Rivas-Ruiz F, Fernandez-Peñas P, Padilla-España L, Sánchez-Blázquez N, Buendía-Eisman A (2017). Sun exposure habits and sun protection practices of skaters. Journal of Cancer Education.

[ref-16] Fitzpatrick TB (1988). The validity and practicality of sun-recreative skin types I through VI. Archives of Dermatological Research.

[ref-17] Glanz K, Gies P, O’Riordan DL, Elliot T, Nehl E, McCarty F, Davis E (2010). Validity of self-reported solar UVR exposure compared to objectively measured UVR exposure. Cancer Epidemiology, Biomarkers & Prevention.

[ref-18] Glanz K, Mayer JA (2005). Reducing ultraviolet radiation exposure to prevent skin cancer, Methodology and measurement. American Journal Preventive Medicine.

[ref-19] Glanz K, Yaroch AL, Dancel M, Saraiya M, Crane LA, Buller DB, Manne S, O’Riordan DL, Heckman CJ, Hay J, Robinson JK (2008). Measures of sun exposure and sun protection practices for behavioral and epidemiologic research. Archives of Dermatological Research.

[ref-20] Green A, Williams G, Neale R, Hart V, Leslie D, Parsons P, Marks GC, Gaffney P, Battistutta D, Frost C, Lang C, Russell A (1999). Daily sunscreen application and betacarotene supplementation in prevention of basal-cell and squamous cell carcinoma of the skin: a randomised controlled trial. The Lancet.

[ref-21] Hall HI, Jorgensen CM, McDavid K, Kraft JM, Breslow R (2001). Protection from sun exposure in U.S: white children aged 6 months to 11 years. Public Health Report.

[ref-22] Hamant ES, Adams BB (2005). Sunscreen use among collegiate athletes. Journal of the American Academy of Dermatology.

[ref-23] International Handball Federation (2018). Beach Handball takes centre stage at Youth Olympic Games. http:/uri/www.ihf.info/en-us/ihfcompetitions/olympicgames/2018youtholympicgames-women/news/newsdetails.aspx?ID=6147.

[ref-24] Jinna S, Adams BB (2013). Ultraviolet radiation and the athlete: risk, sun safety, and barriers to implementation of protective strategies. Sports Medicine.

[ref-25] Lawler S, McDermott L, O’Riordan D, Spathonis K, Eakin E, Leslie E, Gallois C, Bemdt N, Owen N (2012). Relationships of sun-protection habit strength with sunscreen use during outdoor sport and Physical Activity. International Journal of Environmental Research and Public Health.

[ref-26] Lawler S, Spathonis K, Eakin E, Gallois C, Leslie E, Owen N (2007). Sun exposure and sun protection behaviours among young adult sport competitors. Australian and New Zealand of Public Health.

[ref-27] Lee DK (2016). Alternatives to P value: confidence interval and effect size. Korean Journal of Anesthesiology.

[ref-28] Leiter U, Eigentler T, Garbe C (2014). Epidemiology of skin cancer. Advances in Experimental Medicine and Biology.

[ref-29] Molho-Pessach V, Lotem M (2007). Ultraviolet radiation and cutaneous carcinogenesis. Current Problems in Dermatology.

[ref-30] Pagoto S, McChargue D, Fuqua RW (2003). Effects of a multicomponent intervention on motivation and sun protection behaviors among midwestern beachgoers. Health Psychology.

[ref-31] Petty KN, Knee CR, Joseph AK (2013). Susncreen use among recreational cyclists: how intentions predict reported behaviour. Journal of Health Psychology.

[ref-32] Stanton WR, Janda M, Baade PD, Anderson P (2004). Primary prevention of skin cancer: a review of sun protection in Australia and internationally. Health Promotion International.

[ref-33] Toro-Huamanchumo CJ, Burgos-Muñoz SJ, Vargas-Tineo LM, Perez-Fernandez J, Vargas-Tineo OW, Burgos-Muñoz RM, Zentner-Guevara JA, Bada C (2019). Awareness, behavior and attitudes concerning sun exposure among beachgoers in the northern coast of Peru. PeerJ.

[ref-34] World Health Organization (WHO) (2017). Sun protection. Simple precautions in the sun. https://www.who.int/uv/sun_protection/en/.

[ref-35] Wright MW, Wright ST, Wagner RF (2001). Mechanisms of sunscreen failure. Journal of the American Academy of Dermatology.

